# Probiotics' effects on gut microbiota in jaundiced neonates: a randomized controlled trial protocol

**DOI:** 10.3389/fped.2024.1296517

**Published:** 2024-03-08

**Authors:** Chen Jiayi, Wei Jinying, Yuan Yanhan, Liu Tianyu, Chen Juanjuan, Zhang Feng, Fang Xiaohui, Zhang Jinping

**Affiliations:** ^1^ Pediatrics, Shanghai Sixth People’s Hospital Affiliated to Shanghai Jiao Tong University School of Medicine, Shanghai, China; ^2^ College of Food Science and Technology, Shanghai Ocean University, Shanghai, China; ^3^ Pediatrics, Shanghai Sixth People’s Hospital, Shanghai, China

**Keywords:** neonate, gut microbiota, hyperbilirubinemia, phototherapy, probiotic

## Abstract

**Introduction:**

Recent evidence suggests that blue-light phototherapy impacts gut microbiota composition in jaundiced newborns, leading to disturbances closely related to the therapy's side effects. As a result, gut microbiota may serve as a potential intervention target to mitigate these side effects. In this study, we aim to examine the effects of AB-GG (Lactobacillus rhamnosus LGG), Bb-12 (Bifidobacterium animalis Bb-12) and M-16V (Bifidobacterium breve M-16V) and their combination on the intestinal microbiota, metabolomics and phototherapy-related side effects in neonates with jaundice.

**Methods and analysis:**

A total of 100 jaundiced newborns aged two weeks or younger will be included in this randomized, single-blind (the parents knew, but the neonatologists did not know), single-center controlled trial to receive either 10^9^ colony-forming units of AB-GG, Bb-12, M-16V, a combination of the three probiotics with blue-light phototherapy, or blue-light phototherapy alone. The experimental group will be treated with oral probiotics once daily for 30 days, while the control group will receive only blue-light phototherapy. The follow-up duration will last 30 days. The primary outcomes include changes in gut microbiota, metabolomics, and the incidence of phototherapy side effects, assessed after each phototherapy session, as well as on days 10, 20, and 30.

**Ethics and dissemination:**

The study protocol has been approved by the Ethics Committee of our institution. The findings of this trial will be submitted to a peer-reviewed pediatric journal. Its abstracts will be submitted to relevant national and international conferences.

**Clinical Trial Registration:**

http://www.chictr.org.cn/index.aspx, identifer (ChiCTR2000036013).

## Introduction

### Hyperbilirubinaemia and treatments

Hyperbilirubinemia, which manifests as jaundice, is a common benign condition in newborns and one of the leading causes of hospital readmission during the neonatal period (1). It can result from elevated unconjugated or conjugated bilirubin levels. In some infants, jaundice may become severe, with elevated unconjugated bilirubin potentially progressing to acute bilirubin encephalopathy and kernicterus, posing a significant risk of neonatal mortality and long-term neurodevelopmental impairments (2–5). Currently, phototherapy is the most widely used, effective, and safe method to reduce serum bilirubin levels and prevent severe hyperbilirubinemia and bilirubin encephalopathy, and blood exchange therapy may be utilized for patients unresponsive to phototherapy (6, 7).

### Phototherapy and the intestinal microflora

Phototherapy, while effective, is associated with various side effects, such as mother-infant separation, fever, water and electrolyte imbalances, diarrhea, skin damage, bronze baby syndrome, hematological changes, paralytic intestinal obstruction, patent ductus arteriosus, eye damage, and circadian cycle disturbances (8, 9). Research has shown that phototherapy can significantly impact neonatal gut flora and metabolic markers, leading to bacterial flora imbalances that play a crucial role in neonatal jaundice and enterohepatic circulation (10). This disruption may be a significant cause of phototherapy side effects, making gut flora intervention a promising approach to addressing both hyperbilirubinemia and these side effects (11, 12). Previous studies have employed probiotics in treating neonatal hyperbilirubinemia. Probiotics' role in jaundice treatment is mainly related to regulating gut flora, reducing intestinal pH, decreasing β-glucuronidase levels and activity, inhibiting bilirubin enterohepatic circulation, maintaining intestinal motility, promoting bilirubin excretion, and improving feeding tolerance (13–16). Consequently, probiotics represent an alternative therapeutic target for neonatal hyperbilirubinemia, providing a theoretical foundation for using probiotics in the treatment of jaundiced neonates (17, 18).

All three probiotics are among the most extensively studied worldwide and have been certified as safe and effective by the US Food and Drug Administration (FDA) and the European Food Safety Administration (19, 20). Prior research has employed these probiotics in treating various pediatric conditions, such as inflammatory bowel disease, milk protein allergy, and eczema. However, there are limited reports on their use in treating neonatal jaundice, and no systematic clinical research program has been established (21–23). This study aims to investigate the application of these three probiotics in neonatal jaundice treatment and develop a clinical research protocol that may serve as a reference for other neonatal pediatricians.

### Purpose and hypothesis

The objective of this study was to explore whether AB-GG, Bb-12, and M-16V could contribute to neonatal hyperbilirubinemia treatment by regulating gut flora and metabolic imbalances, as well as reducing phototherapy side effects. We hypothesized that children receiving these three probiotics would exhibit a lower incidence of phototherapy side effects. Additionally, we speculated that the experimental group's fecal samples would demonstrate higher diversity and richness in intestinal microbiota compared to the control group. Moreover, we anticipated differences in the dominant bacteria, related metabolites, and metabolic pathways between the experimental and control groups.

## Methods

### Trial design

The study was designed as a randomized, single-blind trial with an allocation ratio of 4:1. (AB-GG combined with phototherapy, Bb-12 combined with phototherapy, M-16V combined with phototherapy, all of the above probiotics combined with phototherapy, or phototherapy alone). The trial was registered at http://www.chictr.org.cn/index.aspx (registration number: ChiCTR2000036013) (24) before enrolling the first participant. Any significant modifications to the trial will be updated on the mentioned website.

### Experiment and subject setup

The trial will be conducted by enrolling newborns admitted to the NICU unit of East Hospital of Shanghai Sixth People's Hospital. As a tertiary hospital, it discharges over 300 newborns annually, including approximately 100 preterm infants and about 150 jaundiced newborns, ensuring a sufficient source of potential participants. All researchers involved have obtained GCP certifications and are either members of the current clinical pediatric team or the pediatric graduate team, guaranteeing the smooth development and execution of basic and clinical trials at any time. The East Hospital of Shanghai Sixth People's Hospital has established a clinical research center and obtained clinical trial qualifications. Recruitment started in June 2021 and will be completed over the following there years.

### Eligibility criteria

(1) Inclusion criteria: (1) Jaundice index: According to the American Academy of Pediatrics phototherapy guidelines, the jaundice index reaches the phototherapy threshold; (2) age ≤2 weeks; (3) Term infants with 37 weeks ≤ gestational age < 42 weeks and 2,500 g ≤ birth weight < 4,000 g; (4) No prior use of antibiotics or ecological agents before specimen collection; (5) Healthy mothers during pregnancy, with no history of special drug use, and no intake of antibiotics or microecological agents before, during, or after childbirth; (6) Enrolled infants were exclusively breastfed, exclusively formula-fed, or mixed-fed before admission; (7) All enrolled infants had neonatal pathological jaundice as defined by “Practical Neonatology” and required hospital admission solely for phototherapy; (8) Informed consent provided voluntarily.
(2) Exclusion criteria: (1) Gestational age <37 weeks or ≥42 weeks; (2) bilirubin levels reaching the exchange blood transfusion standard or elevated direct bilirubin; (3) complications with pneumonia, septicemia, or other diseases; (4) patients with severe immunodeficiency diseases; (5) those with inherited metabolic diseases; (6) congenital biliary malformations or other organ malformations; (7) drug allergies; (8) situations that may warrant exclusion as determined by the researcher, such as a guardian with mental illness or frequent changes in living or working environments, which may result in loss of follow-up.
(3) Elimination criteria: (1) Those refusing the use of their newborn information and their stools and blood; (2) During the treatment process, other drugs might be needed to be added because of other diseases, such as the use of antibiotics and micro-ecological agents.
(4) Criteria for withdrawal from the study: (1) participants who suddenly refuse stool and blood collection and request to withdraw from the study; (2) severe gastrointestinal symptoms, such as intense vomiting, diarrhea, or abdominal distension, and other adverse events that significantly affect the treatment process during the probiotics intervention; (3) neonates requiring exchange blood transfusion due to the continued worsening of jaundice after active phototherapy; (4) neonates lost to follow-up, unreachable, or unable to return to the study center for visits.

### Interventions

(1) Interventions: Neonates will be placed in a blue light treatment box (Ningbo David Medical Device Co., LTD, XHZ model) with the temperature set at 30°C and relative humidity at 55%. Phototherapy eye masks (Foshan Forman Medical Technology Co., Ltd., Yuesun Mechanical Equipment No. 20160015) and phototherapy diapers (Foshan Baojusheng Medical Equipment Co., Ltd., GB/T33280) will be used to cover the eyes and perineum. The treatment will involve LED blue light continuous irradiation with a wavelength of 425–475 nm. All neonates will undergo continuous phototherapy for 12 h, rest for 6–8 h, and then receive another 12 h of continuous phototherapy. The decision to continue with the next 12 h treatment session will depend on the changes in the neonate's jaundice condition.
(2) Study drug and specifications: The patented probiotic combination (contains Bifidobacterium breve 10^9^, Lactobacillus rhamnosus 10^9^ and Bifidobacterium animalis 10^9^ per package) (invention patent, 202,010,414,426.7) will be added to the corresponding groups. The aforementioned drugs are freeze-dried powder, stored at room temperature, with a 30-month shelf life, and manufactured by China Taiwan Jinqiao Biological Co., Ltd. Probiotics intervention: the probiotics will be administered to jaundiced newborns requiring phototherapy. The experimental group will receive the corresponding daily probiotics during phototherapy. The probiotics will be mixed into the milk and consumed once a day, with a treatment course lasting for one month. Clinical study criteria will be controlled to determine the termination of the study.
(3) Criteria for stopping the clinical study: (1) occurrence of serious adverse events occur that may be related to the use of probiotics; (2) if the planned interim analysis achieves the expected difference in efficacy and the intervention is observed to be significantly more effective in the trial group compared to the control group.
(4) Adverse event observation
  (1) Definition of adverse events: In this clinical trial, adverse events are defined as severe vomiting, diarrhea, disruption of normal eating habits, or significant necrotizing enterocolitis occurring during the use of probiotics. Additionally, an adverse event is defined as a severe increase in jaundice value that reaches the index for exchange transfusion.
  (2) Diagnostic criteria of adverse events: (1) Severe vomiting:vomit 5 or more times per day for 2 or more days; (2) Diarrhea 8 or more times per day for 2 or more days; (3) Necrotizing enterocolitis:Neonatal necrotizing enterocolitis is diagnosed according to the Clinical guidelines for the diagnosis and treatment of neonatal necrotizing enterocolitis (2020); (4) According to the Clinical Practice Guideline Revision: Management of Hyperbilirubinemia in the Newborn Infant 35 or More Weeks of Gestation, exchange transfusion criteria have been met.
  (3) Degree of adverse events: (1) Mild: Neonates can tolerate the event, which does not affect treatment, does not require special treatment, and has no impact on the neonates' health. (2) Moderate: Neonates find the event unbearable, affecting the treatment and having a direct impact on their health. (3) Severe: The event endangers the neonate's life, potentially causing death or disability, and requires immediate emergency treatment.
  (4) Possibility of adverse events: Although every bacterium in the probiotic combination used in this study is approved by the National Ministry of Health as safe and reliable, adverse events cannot be completely ruled out. However, the probability of adverse events is expected to be low
  (5) Treatment of adverse events, follow-up methods, and time: In case of adverse events, the incident will be promptly reported, and the clinical trial for the affected neonate will be terminated in a timely manner to prioritize their safety. The patient's stool and gastrointestinal symptoms will be monitored daily, and plain abdominal radiographs will be performed as needed. For patients with severe jaundice requiring exchange transfusion, exchange transfusion therapy will be immediately initiated, as well as monitoring jaundice dynamically and conducting brain MRI and brainstem auditory evoked potential assessments. Post-discharge follow-up will be conducted, selecting various follow-up methods based on the situation. If a neonate with jaundice experiences adverse events after taking probiotics or if such events are suspected to be related to the probiotic administration, strict records should be maintained, the adverse event form should be completed, and the main responsible personnel and the Clinical Research Center of the East Hospital of Shanghai Sixth People's Hospital will be informed within 24 h according to the established process. The ethics committee will decide whether to halt the trial immediately.
  (6) Risk prevention of adverse events: All adverse events occurring during treatment will be graded according to severity and documented in detail using a CRF (Case Report Form). The potential relationship between adverse events and probiotics will be analyzed. If severe adverse reactions occur, the trial should be stopped immediately. Serious adverse events must be reported in strict accordance with the relevant provisions of GCP (Good Clinical Practice). The group will not receive any drugs or additional probiotics in their milk.

### Study procedure

**Figure 1:**
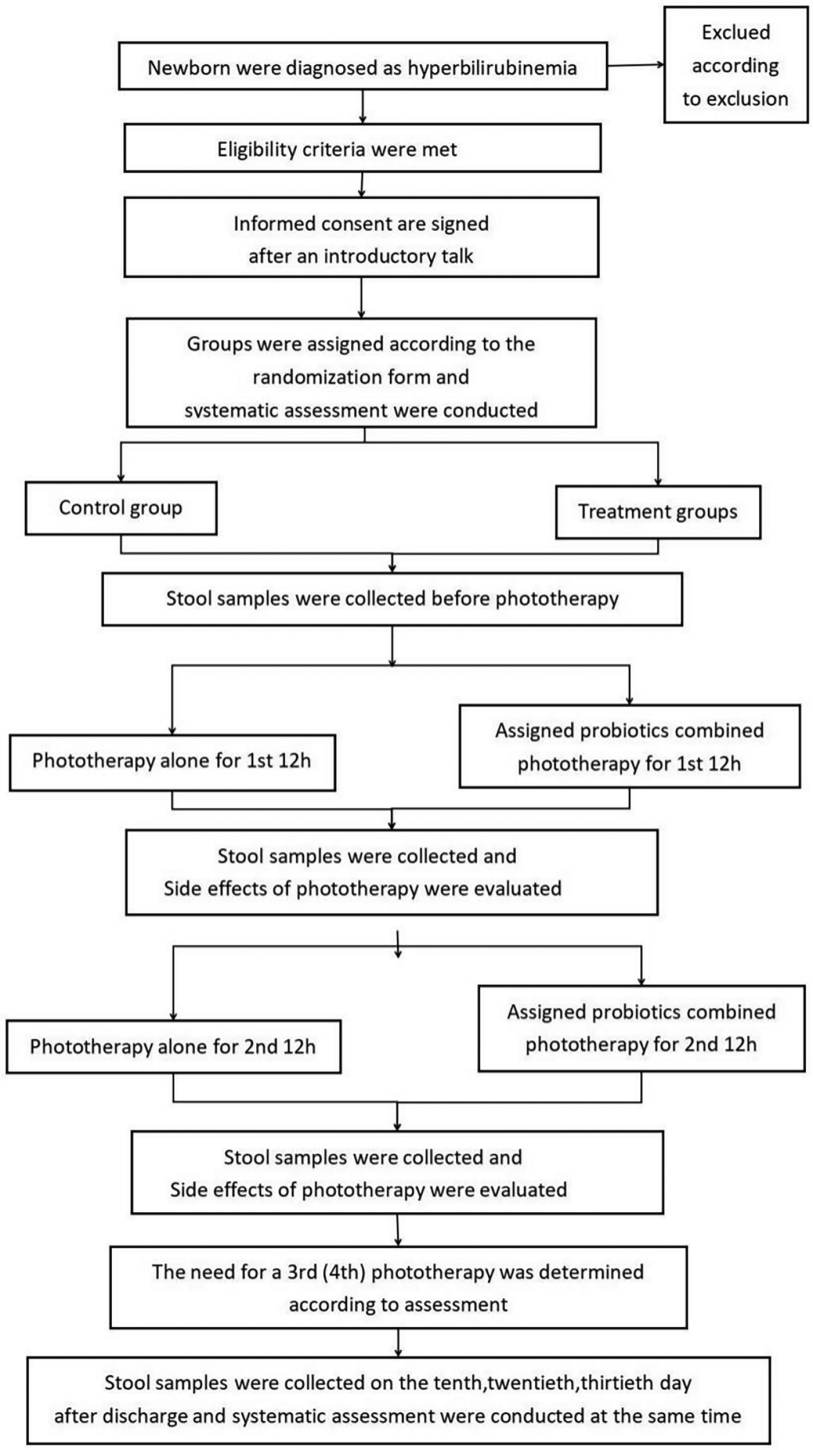
Flow chart of the trial.

Figure 1 illustrates the details of the research procedure. Parents will continue to receive information about the study for 30 days after enrollment when the newborn has confirmed that neonatal hyperbilirubinemia has met the criteria for phototherapy for the appropriate age, including during their regular follow-up visits to the hyperbilirubinemia clinic. Pediatricians participating in the study will sign written informed consent with parents. Subjects will be randomly assigned to receive AB-GG combined with phototherapy, Bb-12 combined with phototherapy, M-16V combined with phototherapy, all of the above probiotics combined with phototherapy, or phototherapy alone, following the method of a digital randomization list. The probiotic dose is 10^9^ colony-forming units (5 drops) administered orally once daily for 1 month. All subjects will be followed up for 1 month after the start of the intervention. Data and specimen collection on days 10, 20, and 30 will be completed during the outpatient follow-up for hyperbilirubinemia.

The arrangements for enrollment during hospitalization and outpatient follow-up after discharge are shown in Table 1. At the beginning of the study, all eligible newborns will undergo a detailed physical examination. The pediatrician will record comprehensive general information (including the mother's prenatal examination, fetal condition, birth history, medication history, feeding history, length, weight, etc.). Jaundice indicators will be recorded before enrollment, blood samples will be collected (for thyroid function and serum bilirubin), and any adverse events will be documented.

**Table 1:** Enrollment and follow-up items during hospitalization and after discharge.

Inspection schedule									
Time window	Enrollment, D0	Phototherapy for 12 h, D1	Rest for 6–8 h after phototherapy, D1	Phototherapy for 12 h, D2	Rest for 6–8 h after phototherapy, D2	Phototherapy for 12 h, D3	Rest for 6–8 h after phototherapy, D3	On the day of discharged, D4	Post-discharge, D10, D20 and D30
Informed consent	X								
Inclusion and exclusion criteria	X	X	X	X	X	X	X	X	X
Feeding situation	X	X	X	X	X	X	X	X	X
Give light therapyintervention		X		X		X			
Random givento probiotics		X		X		X		X	X
check-up	X	X	X	X	X	X	X	X	X
vital sign	X	X	X	X	X	X	X	X	X
Weight/urine output	X	X	X	X	X	X	X		X
Serum examination 1		X							
Systemic assessment	X	X	X	X	X	X	X	X	X
Percutaneous bilirubin determination	X	X	X	X	X	X	X	X	X
Stool assessment andSystemic assessment		X		X		X		X	X

The investigator will re-check the inclusion/exclusion criteria on the first day, perform patient management, conduct detailed physical and serologic examinations prior to phototherapy, and record the adverse events described in Table 1. They will collect stool and blood samples from the subjects. Subjects will receive the first 12 h of phototherapy and the assigned probiotics before phototherapy. After 12 h of phototherapy, researchers will observe phototherapy-related side effects (including body temperature, rash, stool frequency and shape, etc.), record information, collect stool samples, and measure TCB. On the next day, after 6–8 h of rest, the infant will receive a second 12 h phototherapy session while the assigned probiotics are orally administered, and the researchers will again assess and collect fecal samples. The same process will be followed for the third day, and if necessary, subjects may continue with the fourth and fifth 12 h phototherapy sessions, with at least 6 h of rest after each phototherapy session. Stool collection will still be required after each phototherapy session. Subjects will not require further phototherapy and will be discharged once they meet the discharge criteria.

Upon meeting the discharge criteria, the investigator will perform subject management, a detailed physical examination, serological testing, and evaluation of adverse events prior to discharge. Study follow-up on days 10, 20, and 30 will be completed at the outpatient follow-up for hyperbilirubinemia, during which stool and related data will be collected. Infants in the experimental group will continue to receive their assigned oral probiotics during hospitalization after enrollment and during outpatient follow-up for one month.

Stool samples weighing 500 mg and blood samples measuring 0.5 microliters were collected from each subject. The stool samples will be stored at −80°C after freezing, while the blood samples will be tested immediately. There would be no restrictions on the feeding patterns of both the experimental and control groups during the study. Parents are allowed to freely choose between breastfeeding, artificial feeding, and mixed feeding.

### Endpoints

(1) Primary endpoint: significant reduction in the incidence of clinical side effects (i.e., diarrhea, rash, fever, vomiting, convulsions, feeding intolerance, etc.).
(2) Secondary endpoints: after the addition of probiotics, the intestinal flora will be maintained in a stable state after phototherapy, and the changes in metabolic indicators; changes in jaundice (i.e., serum bilirubin, bile acid and transcutaneous bilirubin data), feeding status, changes in urine volume and weight, and vital signs during phototherapy

### Participant timeline

The schedule of enrollment, intervention, assessment, and follow-up of the subjects is shown in Table 1.

### Sample size

In previous clinical trials investigating the effects of probiotics on phototherapy, it was observed that the use of probiotics reduced the incidence of digestive tract side effects caused by phototherapy. Based on the results of a literature review, it was found that the general incidence of such side effects is approximately 47%, and our goal is to reduce this incidence to about 10% by adding the appropriate combination of probiotics. To achieve this goal, a margin of 0.05 was assumed, with a type I error of 0.05 and a power of 0.8. Using a difference test for two-sample ratios, a sample size of 20 cases per group was calculated, resulting in a total of 100 cases in both the experimental and control groups.

### Randomization

(1) The randomized scheme of this experiment will be as follows: 100 cases are planned to be enrolled and divided into 5 groups: C0 (pure phototherapy group), T1 (treatment group 1), T2 (treatment group 2), T3 (treatment group 3) and T4 (treatment group 4), with 20 cases in each group.
(2) The random system parameters will be set as follows:
  • Random system: Taimei Medical eBalance random system
  • Random method: block random
  • Block length: 5
  • Number of blocks: 20
  • Random number: P001–P100.

The random operation steps would be as follows: The random-related parameters are determined according to the project's random scheme. The eBalance stochastic system is configured according to random parameters. The eBalance random system generated a random grouping table according to the configuration (calling SAS software). The eBalance random system exported the random grouping table.

### Blinding

The trial is designed to be single-blind. All neonatologists wouldn't be aware of the group assignments throughout the study, while the parents would be aware of their group assignment to the experimental or control groups.

### Allocation concealment

Based on the randomization table, each enrolled subject will be assigned a serial number in order, which will correspond to one of the following groups: C0 (phototherapy only), T1 (M-16V combined with phototherapy), T2 (Bb-12 combined with phototherapy), T3 (AB-GG combined with phototherapy), and T4 (M-16V combined with Bb-12 combined with AB-GG combined with phototherapy).

### Data collection and management

Each subject will be assigned a unique serial number, and the Case Report Forms (CRFs) will be maintained as a subject's admission history. All fecal microbiota and metabolic results will be entered and stored in a qualified electronic database. The raw materials of CRFs and all study data will be stored in a secure cryptographic cabinet within the research center and will only be accessible to the relevant researchers.

### Monitoring

The results obtained through this study may be published in medical journals, but all trial data and recorded information will be kept confidential in strict accordance with the law. The subjects' personal information will be kept strictly confidential and will not be disclosed unless required by the corresponding law or with their explicit consent. When necessary, the administrative department of the government and the hospital's ethical committee may consult the subjects' data in accordance with the regulations, but only after obtaining appropriate ethical and legal approvals.

This study will develop a data security monitoring plan based on the risk level. All adverse events will be documented in detail and properly handled and followed up until they are resolved or stabilized. Serious adverse events will be promptly reported to the ethics committees, competent authorities, initiators, and drug regulators.

### Statistical analysis

Metagenomic sequencing:Sample extraction and detection: (1) Genomic DNA will be extracted from stool samples using the CTAB method. The quality of the DNA will be analyzed using the Agilent Fragment Analyzer 5,400 automatic capillary electrophoresis system. Library construction and quality control: DNA samples that pass the quality control will be randomly sheared using the Covaris ultrasonic breaker to produce fragments with an average length of approximately 350 bp. (2) These fragments would then be purified and amplified using PCR to generate the final library. Library construction will be performed using the NEB Next®Ultra™ DNA Library Prep Kit for Illumina. Sequencing: The index-coded samples were clustered using the Illumina PE Cluster Kit on the cBot Cluster Generation System. The DNA library was then sequenced using the Illumina Novaseq 6,000 platform to generate 150 bp paired-end reads.

### Statistical analysis and bioinformatics analysis

In SPSS software (version 22.0), chi-square distribution and mean value analysis will be used to analyze the characteristics of subjects. The Mann-Whitney test will be used to compare differences between groups, and the Wilcoxon test will be used to analyze differences in alpha diversity. There are five main steps in bioinformatics analysis:

• Data quality control and de-hosting sequence: KneadData software was used for quality control (based on Trimmomatic) and de-hosting (based on Bowtie2) of original data. FastQC was used to test the rationality and effect of quality control before and after KneadData.
• Species annotation: Kraken2 and self-built microbial nucleic acid database (screening NCBI NT nucleic acid database and RefSeq whole genome database belonging to bacteria, fungi, archaea, and viruses) would be used to calculate the sequence number of the species contained in the sample, and then Bracken will be used to estimate the actual abundance of the species in the sample.
• Common functional database comments: Starting from the quality control and the removal of host gene reads, HUMAnN2 software (based on DIAMOND) will be used to align the reads of each sample to the database (UniRef90). According to the correspondence between UniRef90 ID and each database, we will obtain the annotation information and relative abundance table of each functional database.
• Based on the species abundance table and functional abundance table, PCoA and NMDS dimension reduction analysis (only species) and sample clustering analysis will be analyzed to obtain ɑ diversity, β diversity, bacterial symbiosis network results, etc. When grouping information is available, LEfSe biomarker mining analysis and metabolic pathway comparison analysis can be performed to mine the differences in species composition and functional composition between samples, count metabolite content and screen out characteristic metabolites. Mann-Whitney will be used for assessing gut microbiota and metabolites.
• Annotation of resistance genes: Starting from the clean reads of removing host genes, DIAMOND software will be used to compare and annotate the quality control of each sample and the reads of removing host genes with the antibiotic resistance gene database CARD, and the abundance distribution of resistance genes could be obtained.
• metabolome analysis using the R language MetaboAnalystR package. This package enables the quantification of metabolite content and the identification of characteristic metabolites. MannWhitney U and Kruskal-Wallis tests will be used to analyze differences in gut flora and metabolites between groups.

## Discussion

Neonatal jaundice is a common condition that affects a significant proportion of newborns, with a prevalence ranging from 2.4% to 15% (25). Probiotic supplementation has been shown to improve neonatal jaundice recovery and reduce phototherapy's side effects by regulating bacterial colonies and enhancing immunity (26–28). Previous studies have evaluated the clinical value of probiotic supplementation in treating neonatal jaundice and have provided evidence that combining conventional treatment with probiotic supplementation, including Bifidobacterium, Streptococcus brachii, Chlamydia butyrate, probiotic oligosaccharide, and Bacillus subtilis, significantly improves the therapeutic efficacy of neonatal jaundice (16, 29–32). In addition, probiotic supplementation has been shown to significantly improve neonatal jaundice by reducing total bilirubin levels and accelerating jaundice regression, which can also lead to a reduction in the duration of phototherapy and hospitalization (33–35).

The following limitations of this trial should be considered. First, this study will not include a pure blank control group without phototherapy, and the control group with phototherapy alone will lack data from healthy newborns in the same region and period as a reference. Second, the trial is single-blinded, and the investigators would be aware of the trial-group assignments, which might influence the results. Third, the sample size would be relatively small, the trial will focus on a short observation period with no long-term follow-up of patients, and the study will be conducted only in a single center. Therefore, the methodological quality of clinical trials of probiotic supplementation for neonatal jaundice would need further improvement, and well-designed, larger randomized double-blind trials are necessary to further confirm the efficacy of probiotics.

### Harms

The principal investigators will conduct a weekly review of all adverse events and conduct an investigator meeting when necessary to assess the risks and benefits of the study. The committee will monitor the safety and efficacy of the data to make recommendations for further research.

### Auditing

The hospital ethics committee requires the study to be reviewed every 12 months and may adjust the frequency of reviews based on the actual progress of the study.

## Data Availability

The datasets presented in this article are not readily available because of ongoing research. Requests to access the datasets should be directed to the corresponding authors.
